# Clinical assessment of lateral ankle sprains among Swedish physiotherapists: a nationwide survey comparing practice to international and locally modified frameworks

**DOI:** 10.1186/s13102-025-01505-8

**Published:** 2026-01-08

**Authors:** Isabella Lööf, Farshad Ashnai, Daniel Nygren, Susanne Beischer

**Affiliations:** 1 Unit of Physiotherapy, Department of Health and Rehabilitation, Institute of Neuroscience and Physiology, Sahlgrenska Academy, University of Gothenburg, Gothenburg, Sweden; 2 Sportskadekliniken, Vänersborg, Sweden; 3 Sportrehab Sports Medicine Clinic, Gothenburg, Sweden; 4 Department of Orthopaedics, Institute of Clinical Sciences, Sahlgrenska Academy, University of Gothenburg, Gothenburg, Sweden

**Keywords:** Physiotherapy, Lateral ankle sprain, Clinical assessment, ROAST, Ankle-GO, Return to sport, Patient-reported outcome measures, Evidence-based practice, Sweden, Survey study

## Abstract

**Background:**

Lateral ankle sprains (LAS) are among the most common musculoskeletal injuries globally. Although international frameworks such as the Rehabilitation-Oriented Assessment Tool (ROAST) and Ankle-GO provide structured approaches for LAS assessment and return-to-sport (RTS) evaluation, their implementation in Sweden has not been investigated.

**Methods:**

A cross-sectional online survey was distributed to registered Swedish physiotherapists managing patients with LAS. The survey assessed adherence to the original ROAST, as well as modified versions of the frameworks (ROAST_modified_ and Ankle-GO_modified_). Additionally, physiotherapists’ self-rated confidence in their methods was assessed to explore potential discrepancies between perceived competence and framework adherence. Descriptive and inferential statistics were used to analyse associations between reported assessment practices and physiotherapists’ workplace location, educational level, and clinical experience.

**Results:**

A total of 124 physiotherapists responded. Most (87.1%) reported using fewer than 50% of ROAST criteria, while 48.4% met at least 50% of ROAST_modified_ criteria. Adherence was significantly higher among those working in urban areas (*p* = 0.032), with no significant differences based on educational level or years of clinical experience. Regarding RTS, 36.7% of physiotherapists applied methods aligned with Ankle-GO_modified_, but only 6.5% regularly used patient-reported outcome measures (PROMs) in this context. The most commonly used assessment domains were gait pattern, range of motion, and muscle strength, while PROMs and physical activity level were rarely included.

**Conclusions:**

Adherence to international frameworks for LAS assessment was generally low among Swedish physiotherapists, but higher when modified versions were applied. Urban-based physiotherapists reported higher adherence compared to their rural counterparts, while educational level and experience had little influence. A discrepancy between perceived confidence and actual adherence suggests a gap between self-assessed competence and implementation.

**Supplementary Information:**

The online version contains supplementary material available at 10.1186/s13102-025-01505-8.

## Background

Lateral Ankle Sprains (LAS) are among the most common musculoskeletal injuries encountered by both recreational and professional athletes [1, 2]. A LAS typically occurs when the ankle is forcibly inverted beyond its normal range of motion, potentially leading to ligamentous injury and joint instability [3]. The injury accounts for 20–25% of all musculoskeletal injuries globally [4] and approximately 40% of LAS patients develop chronic ankle instability (CAI) [5] within the first 12 months after injury [6]. Additionally, LAS has the highest recurrence rate among all musculoskeletal injuries of the lower extremities [7]. High recurrence rates may be attributed to inadequate rehabilitation [2] and/or premature RTS [8]. A recent longitudinal study also reported substantial re-injury risk, with 33% of patients sustaining a recurrent LAS within two years [9]. Despite the risks of recurrence and chronic symptoms, RTS often occurs early or without adequate evaluation [5, 10] as LAS is often perceived as a mild and self-healing condition that requires minimal intervention [6].

Physiotherapists play a key role in assessing and treating LAS through evidence-based approaches [11]. The Rehabilitation-Oriented Assessment Tool (ROAST) provides an international framework for LAS evaluation, highlighting ten key domains for assessing mechanical and functional impairments: pain, swelling, range of motions, arthrokinematics, muscle strength, static postural balance, dynamic postural balance, gait, physical activity level, and patient-reported outcome measures (PROMs) [12]. The ROAST aims to help clinicians identify impairments linked to CAI and is intended to guide assessment of both acute LAS and CAI [12].

Return to Sport (RTS) after LAS is a critical phase in the rehabilitation of LAS, as premature return increases the risk of recurrence and the development of CAI [5, 13]. The PAASS framework (Pain, Ankle Impairments, Athlete Perception, Sensorimotor Control, and Sport/Functional Performance) offers a systematic approach to evaluating an athlete’s readiness to RTS. It was developed through a Delphi consensus process involving international experts in ankle injuries, aiming to provide structured guidance in an area where clinical decision-making is often inconsistent. However, its application in clinical settings lacks clear operational guidance [10]. As a compliment, the Ankle-GO protocol was developed to provide a more practical, test-based approach to evaluate readiness for RTS following LAS [14]. It includes four functional performance tests and two patient-reported outcomes, the Foot and Ankle Ability Measure (FAAM) [15] and the Ankle Ligament Reconstruction-Return to Sport after Injury (ALR-RSI) [16]. The functional tests included in Ankle-GO have demonstrated acceptable reliability and validity in prior studies, supporting their use in RTS decision-making [14].

The ROAST framework provides a comprehensive structure for LAS evaluation across ten key domains, and was similarly established through expert consensus to support consistent and evidence-based assessment practices [12]. Although frameworks such as ROAST, PAASS and Ankle-GO offer structured guidance, their implementation in clinical practice is inconsistent [10, 17–21]. A recent French study reported that only a minority of physiotherapists applied ROAST-recommended outcome measures or validated PROMs, highlighting a substantial gap between frameworks and clinical practice [22]. No studies have been identified examining the implementation of these frameworks and tools among Swedish physiotherapists. In Sweden, documented differences in healthcare access exist between urban and rural regions [23], with clinicians in rural areas often working under more limited resources and fewer opportunities for specialized training. Given these contextual differences, disparities in framework adherence might be expected.

Therefore, the aim of this study was to map the assessment methods used by physiotherapists in Sweden for LAS, and compare their reported methods to the international recommendations, including the assessment framework outlined in ROAST and the structured test battery provided in Ankle-GO. Furthermore, the study will explore variations in reported methods based on factors such as location (rural vs. urban) and the physiotherapists’ level of education beyond undergraduate training.

## Methods

### Design

A cross-sectional online survey was conducted to investigate how Swedish physiotherapists assess and manage LAS. The questions of the survey were based on international frameworks ROAST and Ankle-GO, and allowed for wide geographical participation while being time- and cost-efficient [24].

### Survey development

**Fig. 1:**
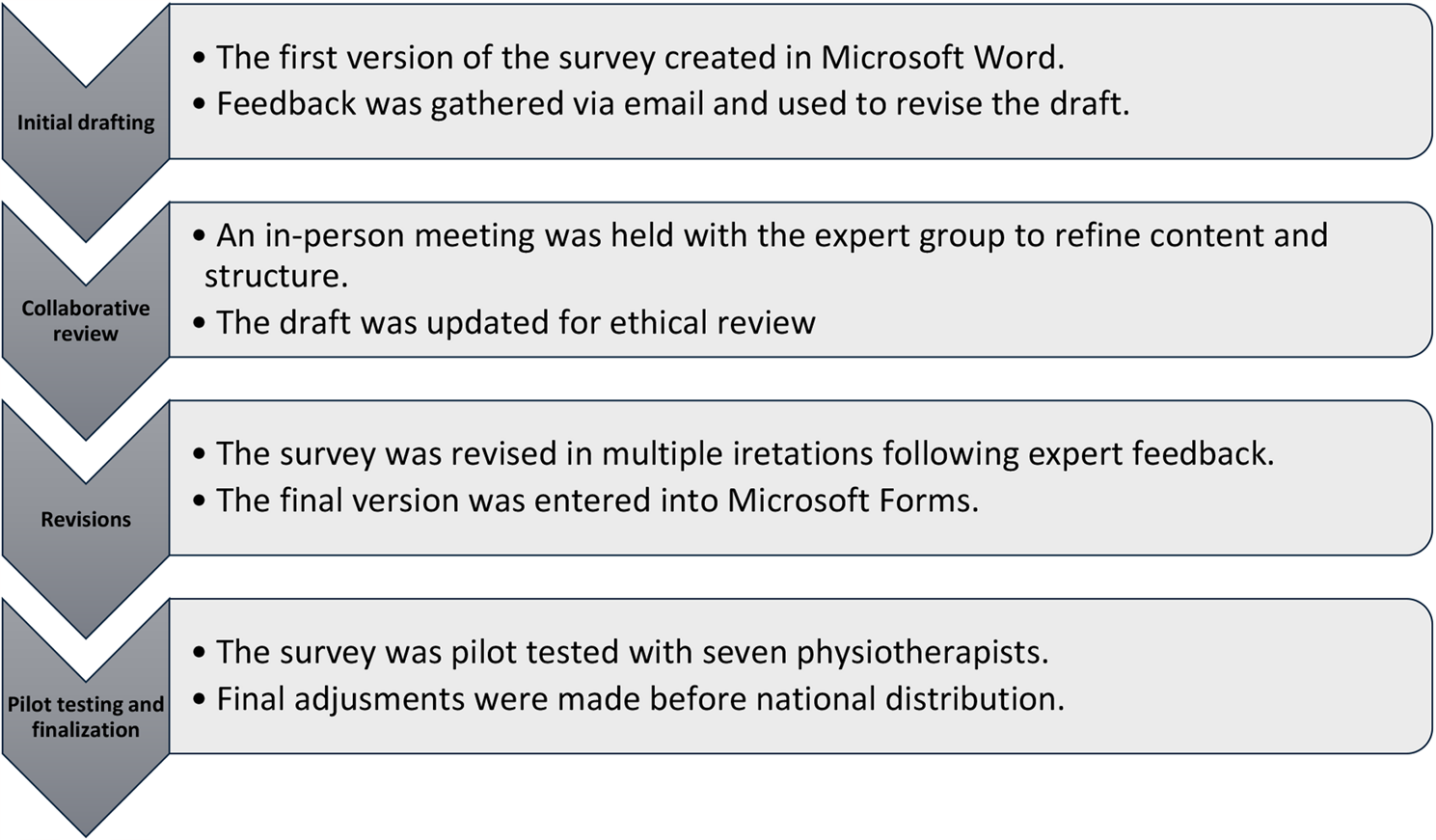
Chronological Overview of the Survey Development Process

The survey was developed through an iterative process by the four authors, who together formed an expert group. All authors are registered physiotherapists with 4 to 17 years of clinical experience and academic backgrounds at bachelor’s, master’s and PhD levels. The first author (IL) drafted the initial versions of the survey, which were subsequently refined based on feedback exchanged through email communications, online meetings, and one in-person session to ensure clarity, clinical relevance and feasibility. The final version was pilot tested by seven clinicians and researchers to evaluate precision and relevance. The survey included multiple-choice, Likert-scale, and open-ended questions. It was distributed via Microsoft Forms starting November 19, 2024. A timeline of the survey development is presented in Fig. 1. The survey is provided in Appendix 1.

The survey included assessment criteria from the original ROAST framework [12] and a modified version (ROAST_modified_), developed by the first (IL) and the last author (SB) to fit Swedish clinical settings where some tools are unavailable. ROAST_modified_ retained core domains but allowed substitution with widely used, valid and reliable local alternatives (e.g., Visual Analogue Scale [VAS] instead of Foot and Ankle Disability Index [FADI] for pain assessment). Open-ended responses were included in the ROAST_modified_ version if they met clinical relevance and validation standards. Following ethical approval, the survey underwent minor refinements based on expert and pilot feedback, limited to clarifying wording and improving the usability of individual items. No changes affecting the substantive content or structure of the survey were made. Table 1 presents a side-by-side comparison of the two versions of ROAST, outlining the specific methods and tools used to assess each domain.

**Table 1:** Comparison of ROAST and ROAST_modified_

	Domain	ROAST	ROAST_modified_
1.	Pain	NRS, FADI [12]	NRS (12), VAS [25]
2.	Swelling	Figure-of-eight method [12]	Figure-of-eight method [12], circumferential measurement directly below both malleoli [26]
3.	Range of Motion	Passive and active range of motion, SEBT [12]	Passive and active range of motion, SEBT [12], goniometry [27], Knee-to-wall test [28]
4.	Arthrokinematics	Posterior talar glide test [12, 29]	Posterior talar glide test [12, 29]
5.	Muscle Strength	Isokinetic dynamometer, handheld dynamometer [12]	Isokinetic dynamometer, handheld dynamometer [12], manual muscle testing [30]
6.	Static Postural Balance	BESS, Foot Lift Test [12]	BESS, Foot Lift Test [12], Single-leg standing [31], Single-leg standing on uneven surface [32]
7.	Dynamic Postural Balance	SEBT, BESS [12]	SEBT, BESS [12], Y–Balance Test [33]
8.	Gait Pattern	Visual observation of gait abnormalities [12]	Visual observation of gait abnormalities [12], recorded video analysis [34]
9.	Physical Activity Level	Tegner Activity Scale, FAAM [12]	Tegner Activity Scale [12], PAI, IPAQ [35], Saltin-Grimby Physical Activity Scale [36]
10.	Patient-Reported Outcome Measure	FAAM, FADI [12]	FAOS, SEFAS [37]

*NRS* Numeric Rating Scale, *FADI* Foot and Ankle Disability Index, *VAS* Visual Analog Scale, *SEBT* Star Excursion Balance Test, *BESS* Balance Error Scoring System, *FAAM* Foot and Ankle Ability Measure, *PAI* Physical Activity Index, *IPAQ* International Physical Activity Questionnaire, *FAOS* Foot and Ankle Outcome Score, *SEFAS* Self–reported Foot and Ankle Score

Similarly, a modified Ankle-GO protocol was used, replacing FAAM and ALR-RSI with Foot and Ankle Outcome Score (FAOS) and Patient Specific Functional Scale (PSFS), as the former have not yet been translated and culturally adapted for a Swedish context. Only the modified version of the Ankle-GO was used in the analysis. Table 2 provides a side-by-side comparison of the two versions.

**Table 2:** Comparison of Ankle-GO and Ankle-GO_modified_

	Ankle-GO	Ankle-GO_modified_
1.	SLS on a flat surface [14]	SLS on a flat surface [14]
2.	mSEBT [14]	mSEBT [14]
3.	SHT [14]	SHT [14]
4.	F8T [14]	F8T [14]
5. & 6.	FAAM, ALR-RSI [14]	FAOS [37], PSFS [38]

*SLS* Single-leg stance test, *mSEBT* Modified Star Excursion Balance Test, *SHT* Side Hop Test, *F8T* Figure-of-8 Test, *FAAM* Foot and Ankle Ability Measure, *ALR-RSI* Ankle Ligament Reconstruction-Return to Sport after Injury, *FAOS* Foot and Ankle Outcome Score, *PSFS* Patient Specific Functional Scale

### Outcome measures

The primary outcome was the degree of alignment between Swedish physiotherapists’ reported assessment methods for LAS and the recommendations outlined in ROAST, ROAST_modified_ and Ankle-GO_modified_. Secondary outcomes included differences in practice patterns based on physiotherapists’ education, clinical experience, and urban (> 200 000 inhabitants) vs. rural (< 200 000 inhabitants) workplace location, as well as inclusion of RTS-phase in rehabilitation. Additionally, physiotherapists’ self-rated confidence in their assessment and evaluation methods for LAS was explored, to assess potential discrepancies between perceived competence and adherence to established frameworks.

### Sampling and sample size

Convenience sampling [39, 40] targeted registered physiotherapists in Sweden treating patients with LAS. Participation was voluntary and anonymous, allowing physiotherapists to respond without their answers being linked to the identities, thereby promoting a higher response rate [39]. An a priori sample size calculation was conducted [41] using G*Power (version 3.1) to determine the minimum number of responses required to statistically detect a 25-percentage point difference with 80% power and a = 0.05. The result indicated that a total of 116 physiotherapists were needed to achieve statistical power.

### Data collection

The survey was distributed at the end of 2024 through professional forums (e.g., Facebook, LinkedIn), physiotherapy associations, email networks, and directly to clinics. Outreach ensured both urban and rural representation across Sweden. The data collection continued until an adequate number of responses was obtained.

### Data analysis

Data was analyzed using the Statistical Package for the Social Sciences (SPSS) (version 29.0.2.0). Descriptive statistics (means, medians, frequencies) were used to provide an overview of the evaluation and assessment of LAS in Sweden [42, 43]. For categorical variables, the number of respondents and their corresponding percentages were reported to illustrate the distribution of responses [44]. Adherence to the ROAST and ROAST_modified_ criteria was summarized as the proportion of respondents fulfilling ≥ 50%, ≥ 70%, or 100% of the criteria. As no established threshold for ROAST has been defined in the literature, the 70% cut-off was selected as a pragmatic operational definition informed by clinical reasoning, corresponding to fulfilling 7 of the 10 criteria and used here to represent satisfactory guideline use.

Chi-square tests [45] were applied to compare framework adherence across education levels and workplace types. Normality was tested with Shapiro-Wilk, and Spearman’s rank correlation was used to assess associations with clinical experience [45]. The significance level for statistical analyses was set to *p* < 0.05.

## Results

### Demographics

A total of 124 physiotherapists responded before the survey was closed on December 4, 2024. The demographic characteristics of the respondents are presented in Table 3.

**Table 3:** Demographic characteristics of responding physiotherapists

	Total (*n* = 124)	Female (*n* = 69)	Male (*n* = 55)
Sex %	-	55.6	44.4
Age (years)^a^	39.1 (11.3)	38.7 (11.8)	39.6 (10.7)
Workplace location			
Urban (> 200 000 inhabitants) %	56.5	62.3	49.1
Rural (< 200 000 inhabitants) %	43.5	37.7	50.9
Clinical experience (years)^b^	11 (5–21.8)	11.5 (5-23.8)	10 (5–20)
Educational level, n (%)			
Bachelor’s degree	77 (62.1)	43 (62.3)	34 (61.8)
One year master’s degree	22 (17.7)	12 (17.4)	10 (18.2)
Master’s degree	18 (14.5)	9 (13)	9 (16.4)
Higher academic education	7 (5.6)	5 (7.2)	2 (3.6)
Specialist physiotherapist, n (%)			
Yes	21 (16.9)	12 (17.4)	9 (16.4)
No	103 (83.1)	57 (82.6)	46 (83.6)

^a^Mean (standard deviation)

^b^Median (Inter quartile range)

### Compliance with the ROAST framework

Table 4 outlines the number of physiotherapists using each assessment method included in the original ROAST and ROAST_modified_ framework. The most frequently used evaluation methods in the original ROAST were related to gait patterns (99.2%), range of motion (80.6%) and pain (63.5%). In contrast, PROMs (0%) and swelling (2.4%) were among the least frequently used methods. Of the 124 respondents, 108 (87.1%) reported using less than 50% of the ROAST criteria, while 16 (12.9%) met at least 50% of the criteria. No respondents reported using 70% or more of the ten ROAST criteria. Physiotherapists working in urban areas were more likely to adhere to at least 50% of the ROAST criteria compared to their rural counterparts (urban: 18.6% [13/70], rural 5.6% [3/54]; *p* = 0.032). No differences in compliance with the ROAST criteria were observed based on educational level (*p* = 0.105).

**Table 4:** ROAST vs. ROAST_modified_

Evaluation Method	ROAST, *n* (%)	ROAST_modified_, *n* (%)
Pain	81 (65.3)	115 (92.7)
Swelling	3 (2.4)	45 (36.3)
Range of Motion	100 (80.6)	122 (98.4)
Arthrokinematics	38 (30.6)	38 (30.6)
Muscle Strength	16 (12.9)	117 (94.4)
Static Postural Balance	7 (5.6)	110 (88.7)
Dynamic Postural Balance	27 (21.8)	53 (42.7)
Gait Pattern	122 (98.4)	123 (99.2)
Physical Activity Level	7 (5.6)	10 (8.1)
PROMs	0 (0)	31 (25)

### Compliance with ROAST_modified_ framework

Among 124 respondents, the most commonly included evaluation methods for LAS were gait patterns (99.2%), range of motion (98.4%), and muscle strength (94.4%), Table 4. In contrast, PROMs (25%) and physical activity level (8.1%) were the least frequently used methods.

Among respondents, 23.4% (*n* = 29) reported using FAOS, and 2.4% (*n* = 3) used Self-reported Foot and Ankle Score (SEFAS). No additional PROMs were identified in the open-ended responses.

Of the 124 respondents, none met all ten ROAST_modified_ criteria, 52 (41.9%) met at least 70%, 60 (48.4%) met at least 50% and 12 (9.7%) met less than 50% of the modified criteria. Physiotherapists working in urban areas were more likely to adhere to at least 50% (urban: 54.3% [38/70], rural: 40.7% [22/54]; *p* = 0.012) and 70% (urban: 42.9% [30/70], rural 40.7% [22/54]; *p* = 0.012) of the ROAST_modified_ criteria compared to their rural counterparts. There was no difference between compliance with ROAST_modified_ criteria and educational level (*p* = 0.093). There was no statistically significant correlation between years of clinical experience and compliance to the ROAST_modified_ criteria (rho = 0.148, *p* = 0.107).

### RTS methods aligned with Ankle-GOmodified protocol

A total of 120 out of 124 physiotherapists reported including the RTS phase at some point during the rehabilitation of LAS. Furthermore, 66.9% of physiotherapists incorporated the RTS-phase for all or the majority of their patients with LAS (see Table 5). Forty-four (36.7%) physiotherapists reported using RTS phase methods aligned with the Ankle-GO_modified_ criteria. No statistically significant difference was found between groups regarding workplace location (urban: 44.1% [30/68], rural 26.9% [14/52]; *p* = 0.053) or between educational level (*p* = 0.406) and adherence to the Ankle-GO_modified_ criteria in those who included RTS phase at some point during the rehabilitation.

**Table 5:** Proportion of physiotherapists including the RTS-phase in the rehabilitation of LAS

Includes RTS-phase in rehabilitation of LAS	Frequency, *n* (%)
Always	31 (25.0)
Yes, for the majority of my patients	52 (41.9)
Yes, for about half of my patients	19 (15.3)
Yes, for less than half of my patients	18 (14.5)
No, I do not include this phase in the rehabilitation	4 (3.2)

Regarding PROMs in RTS assessment, 6.5% (*n* = 8) of respondents reported always using PROMs, while 7.3% (*n* = 9) used them occasionally. The most commonly used PROMs were the PSFS (*n* = 3) and the FAOS (*n* = 6). The remaining respondents who used PROMs did not specify which one they use.

### Self-rated confidence in LAS assessment

**Fig. 2:**
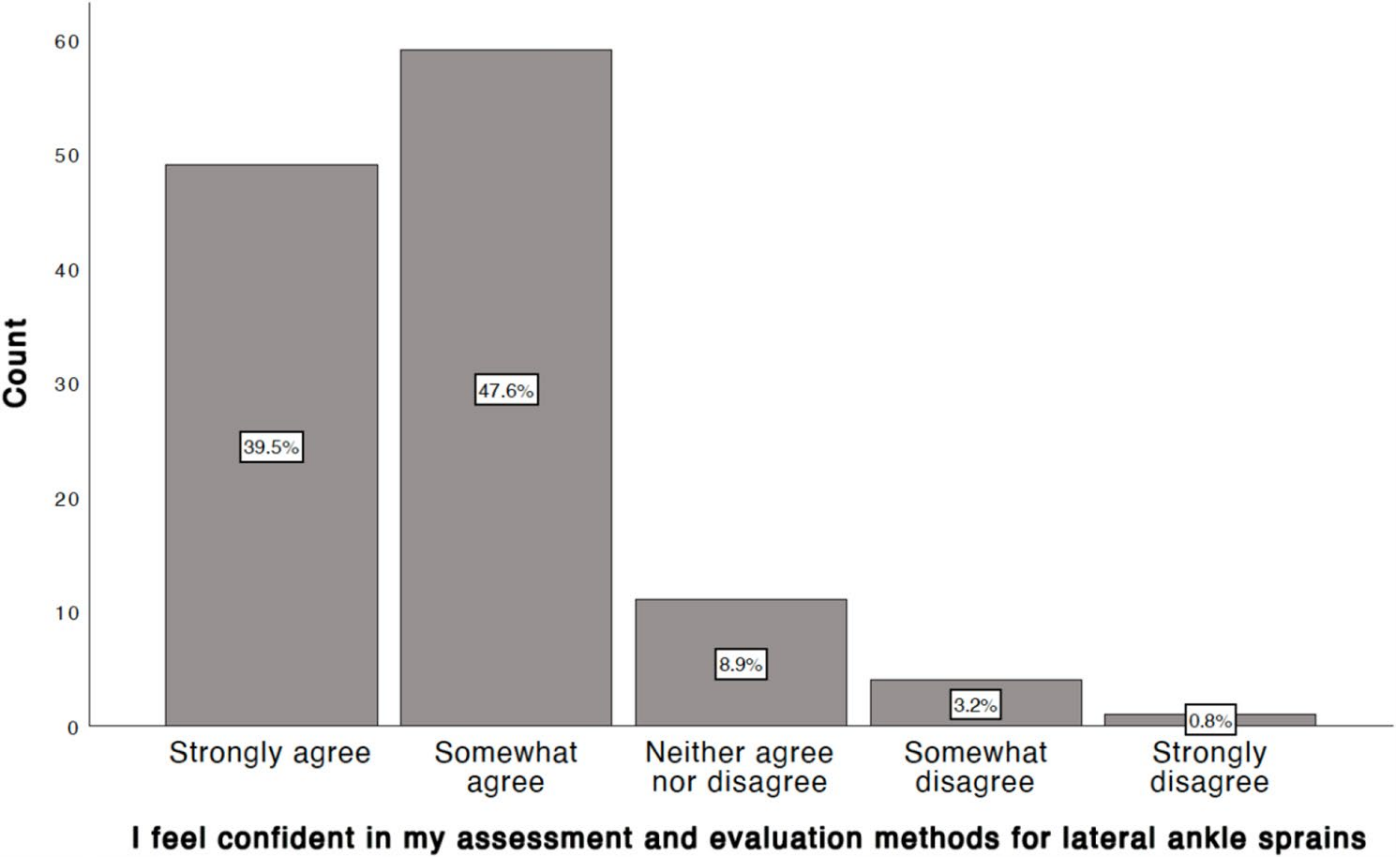
Self-rated confidence in LAS assessment among Swedish physiotherapists

A majority of respondents expressed confidence in their assessment and evaluation methods for LAS (Fig. 2). Specifically, 39.5% strongly agreed and 47.6% somewhat agreed with the statement “I feel confident in my assessment and evaluation methods for lateral ankle sprains.” Five respondents disagreed (1 strongly).

## Discussion

The main findings of this study indicate that overall adherence to the ROAST framework and the Ankle-GO test battery was low among Swedish physiotherapists. Despite using a modified version of ROAST tailored to the Swedish clinical context, fewer than half of the respondents (48.4%) met at least 50% of the ROAST_modified_ criteria. Although overall adherence was low, compliance was markedly higher with the ROAST_modified_ criteria, which allow greater flexibility in the selection of assessment methods. However, increased flexibility alone may not address the underlying causes of low adherence. If clinicians lack of awareness of the frameworks or face practical constraints such as time limitations or limited access to resources, modified versions may not lead to substantial improvements in implementation. This highlights the importance of adapting frameworks to fit the practical realities of clinical practice, especially in settings where rigid adherence may not always be feasible. The underuse of clinical frameworks may relate to limited awareness among clinicians [19–21]. In addition, practical barriers such as time constraints and limited resource have been documented in physiotherapy settings and likely contribute to the low adherence observed [46, 47]. Interestingly, this contrasts with the high proportion of physiotherapists (87.1%) who reported feeling confident in their LAS assessment methods, suggesting a discrepancy between perceived competence and adherence to evidence-based recommendations. Comparable results were reported among French physiotherapists, with low implementation of ROAST and limited use of validated tools highlighting a persistent gap between research and clinical practice [22]. Similar to the present study, respondents reported high confidence in their expertise despite limited adherence to evidence-based recommendations However, since the present survey did not assess physiotherapists’ familiarity with ROAST or Ankle-GO, future research should examine whether increased awareness could enhance adherence and clinical decision-making.

One major barrier to implementing tools such as the ROAST frameworks and the Ankle-GO test battery in Sweden is the lack of translated or culturally adapted assessment methods. Therefore, modified versions of the ROAST and the Ankle-GO were developed and applied in the present study. Despite these modifications the adherence to Ankle-GO criteria was low, suggesting the presence of potential barriers to implementation. This result aligns with earlier studies that report the challenges of using guidelines and complex frameworks in real world settings [17–21, 48, 49]. Further investigation is needed to understand the low adherence to the ROAST and Ankle-GO frameworks, especially in relation to physiotherapists’ clinical routines and perceived relevance. In Sweden, improving adherence may be facilitated through translation, cultural adaptation, and dissemination of currently unavailable assessment tools.

A significant difference in framework adherence was observed between urban and rural physiotherapists, with those practicing in urban areas more likely to meet at least 50% of the criteria for both the ROAST and ROAST_modified_ framework. This disparity could be attributed to differences in access to resources, training opportunities, and professional networks [23]. Previous research has shown that rural healthcare settings often face structural barriers such as limited access to specialists, fewer opportunities for continuing professional development, and reduced interprofessional collaboration, which may influence clinicians’ ability to implement evidence-based practices [50, 51]. Although the difference between workplace location (urban 44.1% versus rural 26.9%) was not statistically significant (*p* = 0.053), the observed trend between groups, with greater alignment with the Ankle-GO_modified_ test battery among urban physiotherapists, may warrant further investigation. There is also evidence suggesting that musculoskeletal care delivered in rural settings may include a higher proportion of low-value or non-evidence-based interventions, potentially reflecting contextual constraints rather than clinician preference [52]. Rural physiotherapists may benefit from additional support to improve adherence to evidence-based frameworks and help reduce urban-rural disparities. The final sample of 124 respondents exceeded the 116 estimated from the a priori sample size calculation. However, a larger sample could have enabled more robust subgroup analyses to further explore observed trends. Future research should aim to explore these potential influences, including the availability of resources, training opportunities, and professional networks, and may benefit from larger sample to further investigate the observed trend.

Another notable finding was the low use of PROMs in this study. Even within the ROAST_modified_ criteria, only 25% of physiotherapists reported using them. Similar underuse and implementation challenges have been reported elsewhere [53, 54]. Possible barriers include time constraints, unfamiliarity, and perceived complexity, which have also been reported in physiotherapy settings [46, 47, 55]. This raises the concern that key biopsychosocial factors, such as fear of reinjury or readiness to RTS, may be overlooked, despite their known importance for recovery [56]. Although Ankle-GO incorporates an element of psychological readiness, it does not fully capture broader psychosocial domains such as fear of movement, coping, or overall well-being. Tools that integrate both physical and psychological readiness may therefore support a more holistic approach. As the biopsychosocial model gains prominence in rehabilitation [57], future research should explore how PROMs, particularly those capturing psychosocial aspects of recovery and well-being, can be better integrated into practice.

The online survey allowed for broad and diverse participation, with accessibility and anonymity likely improving response rates and reducing bias [39, 58]. However, distribution via social media and professional networks may have introduced selection bias, potentially overrepresenting physiotherapists with an interest in sports medicine or research [58, 59]. As a result, adherence rates in this sample may be higher than in the broader physiotherapy population, particularly among those in general practice who may not routinely use LAS-specific frameworks.

The content validity of the survey was strengthened by an iterative development process [60], which included feedback from experienced physiotherapists and a pilot survey. However, despite these efforts, internal validity may have been affected by respondents’ interpretation of the questions, particularly those related to the clinical decision-making [24]. Variation in physiotherapists’ backgrounds, experiences, and interpretations may have contributed to response variability, particularly for more subjective items [61]. While Likert-scale items help standardize responses to some extent, qualitative responses may have introduced variability [62]. To gain a deeper understanding of physiotherapists’ perspective on LAS rehabilitation and RTS, future research may benefit from incorporating qualitative methods. Such approaches can reveal contextual factors and unmet clinical needs that are not easily captured through surveys [63].

A strength of the study was the relatively large number of respondents, supported by an a priori sample size calculation that ensured sufficient power to detect meaningful differences in physiotherapy practices. The development of modified versions of the ROAST framework and the Ankle-GO test battery to the Swedish clinical context, due to limited availability or use of certain assessment tools, was conducted based on expert discussions and pilot testing. While this process enhanced the clinical applicability of the frameworks and maintained alignment with core assessment principles, the modified versions have not undergone formal validity or reliability testing, and their measurement properties therefore remain unknown. This should be considered when interpreting the results, as the lack of formal validation may affect both the precision and comparability of our findings. In addition, the modifications may limit the comparability with studies conducted in other contexts. Future studies should therefore evaluate the measurement properties of these modified versions to strengthen their utility in both research and clinical practice.

Several potential limitations should be acknowledged. Despite mandatory questions, complex or subjective items may have prompted rushed or incomplete responses, affecting data quality [64]. Non-responses to open-ended questions may also have limited qualitative depth and reduced effective sample size, impacting generalizability [65]. Furthermore, anonymous data collection, while ethically advantageous and likely encouraging participation, prevented follow-up or clarification of unclear answers [66]. However, these methodological limitations are unlikely to have substantially influenced the overall patterns observed in this study.

### Clinical implications

The findings of this study highlight the need for targeted strategies to strengthen the implementation of evidence-based LAS assessment within the Swedish physiotherapy context, particularly in rural areas. Increasing awareness of available assessment frameworks and providing accessible training, both within undergraduate programmes and through continuing professional development, may support more consistent clinical practice. Digital formats such as online courses, recorded lectures, and virtual peer-learning groups could facilitate participation among physiotherapists in rural regions with limited access to in-person training.

Supporting the use of adapted frameworks such as ROAST_modified_ may also help clinicians integrate structured clinical reasoning activities, for example through case discussions or reflective exercises, thereby supporting critical evaluation of assessment choices and alignment with current evidence. Continued efforts to translate, adapt and disseminate validated assessment tools are essential for broader uptake in Swedish physiotherapy.

## Conclusions

Adherence to international frameworks for LAS assessment was generally low among Swedish physiotherapists, but higher when modified versions were applied. Urban-based physiotherapists reported higher adherence compared to their rural counterparts, while educational level and experience had little influence. A discrepancy between perceived confidence and actual adherence suggests a gap between self-assessed competence and implementation.

## Supplementary Information

Supplementary Material 1: Appendix 1 – The survey.

## Data Availability

The data that support the findings of this study are available from the corresponding author, upon reasonable request.

## Abbreviations

ALR-RSI: Ankle Ligament Reconstruction-Return to Sport after Injury
ATFL: Anterior Talofibular Ligament
BESS: Balance Error Scoring System
CAI: Chronic Ankle Instability
CFL: Calcaneofibular Ligament
F8T: Figure-of-8 Test
FAAM: Foot and Ankle Ability Measure
FADI: Foot and Ankle Disability Index
FAOS: Foot and Ankle Outcome Score
IPAQ: International Physical Activity Questionnaire
LAS: Lateral Ankle Sprains
mSEBT: Modified Star Excursion Balance Test
NRS: Numeric Rating Scale
PAI: Physical Activity Index
PROMs: Patient-Reported Outcome Measures
PSFS: Patient-Specific Functional Scale
ROAST: Rehabilitation Assessment Tool
RTS: Return To Sport
SEBT: Star Excursion Balance Test
SEFAS: Self–reported Foot and Ankle Score
SFAIM: Svensk förening för Fysisk Aktivitet och Idrottsmedicin
SHT: Side Hop Test
SLS: Single-leg stance test
SPSS: Statistical Package for the Social Sciences
VAS: Visual Analog Scale
