# Supplementary material for: Clinical assessment of lateral ankle sprains among Swedish physiotherapists: a nationwide survey comparing practice to international and locally modified frameworks
Source: BMC Sports Sci Med Rehabil. 2026 Jan 8;18:61. doi: 10.1186/s13102-025-01505-8 (PMC12874988; doi:10.1186/s13102-025-01505-8)
Supplement: Supplementary file 1 — Supplementary Material 1: Appendix 1 – The survey. [file 13102_2025_1505_MOESM1_ESM.docx]

# Appendix 1 – The Survey (English Translation)

Hello,

Would you be able to spend about 15 minutes completing this survey?
The survey is aimed at physiotherapists who have experience treating patients with lateral ankle sprains. Participation is voluntary and completely anonymous.

The aim of this survey study is to improve understanding of which methods are used for this injury and to identify areas where clinical care may be improved. The study will be presented as a master's thesis at the University of Gothenburg and submitted as a scientific article.

Thank you in advance!

**1. Gender:**
- Female
- Male
- Prefer not to say / Not specified

**2. Age:**

**3. What type of workplace do you work at?**
- Public primary care
- Private primary care with regional agreement
- Private primary care without regional agreement
- Public specialized care (hospital)
- Private specialized care (hospital)
- Other

**4. How long have you worked as a physiotherapist? (years)**

**5. Do you work in an urban area (>200,000 inhabitants) or a rural area (<200,000 inhabitants)?**
- Urban
- Rural

**6. What level of education in physiotherapy do you have?**
- Bachelor's degree
- Master's degree (1 year)
- Master's degree (2 years)
- Higher academic degree

**7. Are you a certified specialist physiotherapist?**
- Yes
- No

**8. If yes – in which area(s) are you specialized? (You may select more than one)**
- Orthopaedics
- Physical activity and sports medicine
- OMT (Orthopaedic Manual Therapy)
- Other (please specify):

**9. Approximately how many patients with lateral ankle sprains do you treat per year?**
- None
- 1–10
- 11–20
- 21–40
- More than 40

**10. When do you believe the assessment of a lateral ankle sprain should be performed to ensure the most reliable examination?**
- Immediately
- After 1–3 days
- After 4–7 days
- After more than 7 days

**11. Which specific tools do you use to assess lateral ankle sprains? (Multiple answers allowed)**
- Ottawa Ankle Rules (OAR)
- Bernese Ankle Rules (BAR)
- Grading scale for ligament injuries (Grade I, II, III)
- Other (please specify)
- I do not use specific criteria for this

**12. Which specific stability tests do you use to assess lateral ankle sprains? (Multiple answers allowed)**
- Anterior drawer test
- Talar tilt test
- Inversion test
- Cotton test
- Compression test
- I do not use specific stability tests for assessing lateral ankle sprains

**13. How do you assess pain in patients with lateral ankle sprains? (Multiple answers allowed)**
- Numeric Rating Scale (NRS), e.g. 0–10
- Visual Analog Scale (VAS)
- Other (please specify)
- I do not assess pain in these cases

**14. How do you assess swelling in patients with lateral ankle sprains? (Multiple answers allowed)**
- Figure-of-eight method
- Circumference measurement directly below the malleoli
- Visual observation
- Other (please specify)
- I do not assess swelling in these cases

**15. How do you assess range of motion in patients with lateral ankle sprains? (Multiple answers allowed)**
- Passive range of motion (PROM)
- Active range of motion (AROM)
- Goniometry
- Knee-to-wall test
- Star Excursion Balance Test (SEBT)
- Other (please specify)
- I do not assess range of motion in these cases

**16. Talocrural arthrokinematics refers to small gliding movements between talus, tibia, and fibula during dorsiflexion or plantarflexion. How do you assess this in patients with lateral ankle sprains? (Multiple answers allowed)**
- Posterior talar glide test
- Other (please specify)
- I do not assess arthrokinematics in these cases

**17. How do you assess ankle muscle strength in lateral ankle sprain patients? (Multiple answers allowed)**
- Isokinetic dynamometer
- Handheld dynamometer
- Manual muscle testing (gross strength)
- Other (please specify)
- I do not assess muscle strength

**18. How do you assess static postural balance in patients with lateral ankle sprains? (Multiple answers allowed)**
- Single-leg stance (e.g. <30 seconds)
- Single-leg stance on uneven surface
- Foot Lift Test
- Romberg test
- Berg Balance Scale (BBS)
- Balance Error Scoring System (BESS)
- Other (please specify)
- I do not assess static balance

**19. How do you assess dynamic postural balance? (Multiple answers allowed)**
- Star Excursion Balance Test (SEBT)
- Balance Error Scoring System (BESS)
- Berg Balance Scale (BBS)
- Tandem gait
- Y-Balance Test
- Other (please specify)
- I do not assess dynamic balance

**20. How do you assess the patient's gait pattern? (Multiple answers allowed)**
- Visual observation
- Video analysis
- Other (please specify)
- I do not assess gait pattern

**21. How do you determine the patient's physical activity level prior to injury? (Multiple answers allowed)**
- Tegner Activity Scale
- Physical Activity Index (PAI)
- International Physical Activity Questionnaire (IPAQ)
- Saltin-Grimby Physical Activity Scale
- Clinical interview/anamnesis
- Other (please specify)
- I do not assess physical activity level

**22. Which Patient-Reported Outcome Measures (PROMs) do you use to assess lateral ankle sprains? (Multiple answers allowed)**
- Foot and Ankle Outcome Score (FAOS)
- Self-reported Foot and Ankle Score (SEFAS)
- Other (please specify)
- I do not use PROMs

**23. Do you include the return to participation phase in rehabilitation?**
- Always
- For the majority of my patients
- For about half of my patients
- For fewer than half
- No, I do not include this phase

**24. If yes – how long does it usually take until return to participation?**
- X weeks

**25. Do you include the return to sport phase in rehabilitation?**
- Always
- For the majority of my patients
- For about half of my patients
- For fewer than half
- No, I do not include this phase

**26. If yes – how long does it usually take until return to sport?**
- X weeks

**27. Do you use specific tools to assess readiness to return to sport?**
- Yes
- Sometimes
- No

**28. If yes or sometimes – Which balance tests do you use?**
- SEBT
- Single-leg stance test (SLS)
- Other (please specify)
- I do not use balance tests for return to sport

**29. Which hop tests do you use? (Multiple answers allowed)**
- Vertical jump test
- Single-leg hop for distance
- Square hop test
- Side hop test (SHT)
- Figure-of-8 test (F8T)
- Triple hop test
- Drop jump
- Hopping (bilateral/unilateral)
- Crossover hop for distance
- Other (please specify)
- I do not use hop tests

**30. Do you use PROMs for return to sport?**
- Yes
- Sometimes
- No

**31. If yes or sometimes – Which PROMs do you use?**
- Free text

**32. Do you include the return to performance phase?**
- Always
- For the majority of my patients
- For about half
- For fewer than half
- No

**33. If yes – how long does it usually take until return to performance?**
- X weeks

**34. Do you recommend analgesics?**
- Yes
- Sometimes
- No

**35. If yes – Which ones?**
- NSAIDs (e.g. ibuprofen, ASA, naproxen)
- Paracetamol (e.g. acetaminophen)
- Both

**36. Do you use functional support (e.g. brace or tape) to enable early loading?**
- Yes, often
- Yes, sometimes
- No

**37. What type of rehabilitation do you mainly use?**
- Supervised rehab with physiotherapist
- Home exercise programs
- Combination
- Other (please specify)
- None of the above

**38. How often do you include patient education?**
- Very often
- Often
- Sometimes
- Rarely
- Never

**39. How long do you usually follow patients with lateral ankle sprains?**
- Less than 1 week
- 1 to <2 weeks
- 2 to <4 weeks
- 1 to <2 months
- 2 to <3 months
- 3 to <4 months
- 4 to <5 months
- 5 to <6 months
- More than 6 months

**40. When is treatment completed?**
- When patient walks without aid
- Return to everyday physical activity
- Return to sport
- Return to performance
- Other (please specify)

**41. Please indicate your agreement: “I feel confident in my assessment and evaluation methods for lateral ankle sprains.”**
- Strongly agree
- Somewhat agree
- Neutral
- Somewhat disagree
- Strongly disagree

**42. What kind of continuing education activities do you participate in? (Multiple answers allowed)**
- Courses, workshops
- Conferences, seminars
- Online training, webinars
- Clinical mentorship, supervision
- Independent literature review
- Other
- I do not participate in further training on this topic

**43. How often do you update your knowledge and practice regarding ankle sprain rehabilitation?**
- Daily
- Weekly
- Monthly
- Quarterly
- Yearly

**Thank you for your participation!**
